# β_2_-Adrenergic receptor promoter haplotype influences the severity of acute viral respiratory tract infection during infancy: a prospective cohort study

**DOI:** 10.1186/s12881-015-0229-3

**Published:** 2015-09-14

**Authors:** Pingsheng Wu, Emma K Larkin, Sara S Reiss, Kecia N Carroll, Marshall L Summar, Patricia A Minton, Kimberly B Woodward, Zhouwen Liu, Jessica Y Islam, Tina V Hartert, Paul E Moore

**Affiliations:** Center for Asthma & Environmental Health Sciences Research, Department of Medicine, Division of Allergy, Pulmonary and Critical Care Medicine, Suite 6100 Medical Center East, Nashville, TN 37232 USA; Department of Pediatrics, Vanderbilt University School of Medicine, 313 Oxford House, Nashville, TN 37232 USA; Department of Genetics and Metabolism, Division of Genetics and Metabolism, Children’s National Health System, 111 Michigan Avenue, NW, Washington, DC 20010 USA; Department of Biostatistics, Vanderbilt University School of Medicine, 2525 West end Ave, Suite 1100, Nashville, TN 37203 USA; Department of Epidemiology, Gillings School of Global Public Health, University of North Carolina, Chapel Hill, NC 27599 USA; Division of Pediatric Allergy, Immunology and Pulmonary, Department of Pediatrics, Vanderbilt University School of Medicine, 2200 Children’s Way, 11215 Doctor’s Office Tower, Nashville, TN 37232 USA

## Abstract

**Background:**

Despite the significant interest in β_2_-Adrenergic receptor (ADRB2) polymorphisms related to asthma, whether ADRB2 genetic variants are similarly associated with acute respiratory tract infections have not been studied. We hypothesized that genetic variants in ADRB2 associated with a response to asthma therapy during an asthma exacerbation were also associated with severity of acute respiratory tract infections.

**Methods:**

To test this hypothesis, we genotyped 5 common polymorphisms in the promoter region and coding block of the ADRB2 gene (loci -2387, -2274, -1343, +46, and +79) from 374 Caucasian and African American term infants who were enrolled at the time of acute respiratory illness over four respiratory viral seasons. Severity of respiratory tract infections was measured using a bronchiolitis severity score (BSS; range = 0-12, clinically significant difference = 0.5) with a higher score indicating more severe disease. We assigned the promoter, coding and combined promoter and coding haplotypes to the unphased genotype data. The associations between each of these five single-nucleotide polymorphisms (SNPs) as well as the haplotypes and infant BSS were analyzed using nonparametric univariate analysis and multivariable proportional odds model separately in Caucasians and African Americans.

**Results:**

There was no significant association between infant BSS and each of the SNPs in both Caucasians and African Americans. However, promoter haplotype CCA was associated with a decreased BSS in African Americans in a dose dependent manner. The median (interquartile range) BSS of infants with no copies of the CCA haplotype, one copy, and two copies of the CCA haplotype were 5.5 (2.0, 8.0), 4.0 (1.0, 7.5), and 3.0 (1.0, 4.0), respectively. This dose dependent relationship persisted after adjusting for infant age, gender, daycare exposure, secondhand smoke exposure, prior history of breastfeeding, siblings at home, and enrollment season (adjusted odds ratio: 0.59, 95 % confidence interval: 0.36, 0.98). There was no similar protective relationship of haplotype CCA on severity of respiratory tract infections identified in Caucasians.

**Conclusions:**

ADRB2 genotype may be predictive of severity of acute respiratory tract infections in African Americans, and potentially identify a subset of infants who may respond to beta-agonist therapy.

**Electronic supplementary material:**

The online version of this article (doi:10.1186/s12881-015-0229-3) contains supplementary material, which is available to authorized users.

## Background

Respiratory tract infection is common in infants and young children and is a major public health problem in this age group [1]. It is mainly caused by infection of seasonal viruses, such as respiratory syncytial virus (RSV) and rhinovirus [2–4]. Most infections manifest as an upper respiratory infection, while 20–40 % develop lower respiratory infection, and a small number (2 – 3 %) of infected infants require hospitalization [5, 6]. This is of particular concern as infants with lower respiratory viral infection are at increased risk of developing recurrent wheezing or childhood asthma [7–13]. Several risk factors have been identified for the development of severe lower respiratory viral infections, such as premature birth, young age, being born in relation to the RSV season, underlying chronic lung disease, congenital heart disease, high parity, young maternal age and poor socioeconomic factors [14–16]. Recently, genetic polymorphisms have been identified which are associated with severe viral infection [17, 18]. Many of the identified genetic variants associated with severe lower respiratory tract infections are also associated with and pathways common to asthma, including innate immune genes involved in cytokine and chemokine signaling, and epithelial cell function [13, 17, 19–23].

As the clinical hallmark of asthma is wheezing, and therapy includes beta agonists, a number of clinical studies over the past 2 decades have examined the relationship between genetic variants in β_2_-Adrenergic receptor (ADRB2) and asthma [24–30]. The polymorphism that resulted in the Gly16 allele was associated with decreased bronchodilator responses in early clinical studies; however, larger clinical studies have provided equivocal results [31–33]. Other genetic variants in the promoter region of ADRB2 have also been associated with asthma severity [28, 34]. Despite the significant interest in ADRB2 polymorphisms related to asthma, the relative contribution of ADRB2 genetic variants to severity of respiratory viral infections has not been established. As wheezing is also the clinical hallmark of lower respiratory viral infections, and variable response to bronchodilators has been demonstrated in infants hospitalized with RSV lower respiratory viral infections, it is possible that ADRB2 genetic variants also contribute to the severity of acute respiratory viral infections [35, 36].

The aim of the present study was to explore associations between the genetic variants in ADRB2 that have been associated with a response to asthma therapy during an acute asthma exacerbation and the severity of infant acute respiratory viral infections. We examined the relationship between 5 common polymorphisms in the promoter region and coding block of the ADRB2 gene and infant severity of respiratory infections in a prospective cohort of Caucasian and African American infants.

## Methods

### Study population

In order to examine the association of genetic variants in the regulatory regions of the ADRB2 gene and the severity of viral respiratory infections during infancy, we conducted an analysis of infants enrolled in the Tennessee Children’s Respiratory Initiative (TCRI), a prospective cohort of mother-infant dyads enrolled during an infant’s acute viral respiratory illness over four respiratory viral seasons, 2004–2008. Term (≥37 weeks) and non-low birth weight (≥2275 g) infants who were otherwise healthy with no significant co-morbidities or cardio-pulmonary disease were eligible, with oversampling for hospitalization infants. Eighty five percent of enrolled subjects had at least one respiratory virus identified by polymerase chain reaction (RSV, rhinovirus, influenza, parainfluenza, Human Metapneumovirus, and coronavirus); 56 % had lab-confirmed RSV infection. The rationale, methods, and detailed inclusion and exclusion criteria have been reported previously [37]. Although 630 infants were enrolled in TCRI, this study was limited to the 261 Caucasian and 113 African American infants for whom DNA was available. The protocol was approved by the Institutional Review Board of Vanderbilt University, and mothers provided informed consent for their infants. All parents provided written informed consent for both their and their child’s study participation.

### Infant acute respiratory infection severity

Infants included in this study had either upper respiratory tract infections or lower respiratory tract infections. The severity of acute respiratory infection was determined by experienced research nurses through medical record chart review using an ordinal bronchiolitis severity score (BSS). The score is an aggregate of assigned values ranging of 0–3 in categories of respiratory rate, room air oxygen saturation, the presence and extent of wheezing, and the presence and extent of flaring and retractions [37–39]. The BSS ranges from 0 to 12, with higher scores indicating more severe illness and a difference of 0.5 being a clinically significant difference [40]. We measured the BSS at the end of the outpatient visit or after discharge from an inpatient admission, and the most severe recorded value was used for analysis.

### Genotype determination

DNA was extracted by Vanderbilt’s DNA core laboratory from whole blood or saliva samples (Oragene by DNA Genotek) by standardized protocols. All oragene samples were processed immediately after collection, while blood samples were either processed immediately or frozen before processing. Genotyping at five pre-specified loci (-2387, -2274, -1343, +46, and +79) of ADRB2 gene was done using Taqman SM© Genotyping Assays (Applied Biosystems, Foster City, CA, USA) with predetermined primers. The five single-nucleotide polymorphisms (SNP)s were rs1432623, rs11168068, and rs2400707 at the promoter region, and rs1042713 SNP and rs1042714 SNP at the coding region of ADRB2 gene. Probes for the Applied Biosystems assays used were labeled at their 5′end with either VIC® or 6-carboxy-fluorescine (FAM) reporter dyes. The reaction components include: 2.5 μl Taqman Universal PCR Master Mix (Applied Biosystems), 0.125 μl or 0.250 μl assay mix (Applied Biosystems) and approximately 5 ng of genomic DNA in a total volume of 10 μl per single tube reaction. PCR amplification was performed in a thermal cycler (Techne TC-412) with an initial step of 95 °C for 10 min followed by 50 cycles of 92 °C for 15 s and 60 °C for 1 min. After amplification, the fluorescence of each sample was read on the ABI 7900HT (DNA Resources Core at Vanderbilt University) and analyzed with the Sequence Detection Software (Applied Biosystems). Five percent of samples were run as blind duplicates with 100 % concordance. Primers and probes for all SNPs are listed in Table 1.

**Table 1:** Primers and probes of the 5 ADRB2 SNPs

Loci	rs number	Primers	Probes
−2387	rs1432623	Forward Primer	Vic- TCACACAAGTATAGTTTG
		5′-TTCTAAACCACTAAGTAATTTATGTAAACTTCGCTT-3′	
		Reverse Primer	Fam- CACACAAGTGTAGTTTG
		5′-GGTAAGCAAGAATTGAATGATATAGTAAGAAATATGAAAA-3′	
−2274	rs11168068	Forward Primer	Vic- AATCACGAAGTACCTGATTT
		5′-GGAAGTGACTTTATGCCCCTTTAGA-3′	Fam- TCACGAAGTACCTAATTT
		Reverse Primer	
		5′-AGATTCACCAAACTTGGAGCTTTCT-3′	
−1343	rs2400707	Forward Primer	Vic-TTCACATGGCACAACC
		5′-TAAGTCACAG ACGCCAGATGGT-3′	Fam- CACATGGCGCAACC
		Reverse Primer	
		5′-AACAAA CTATCCAGCA GATGAAAGGA T-3′	
+16	rs1042713	Forward Primer	Vic- CAC CCAATGGAAGCC
		5′- CGGCAGCGCCTTCTTGCTGGCAC-3′	Fam-CAC CCAATAGAAGCC
		Reverse Primer	
		5′-TGCGTGACGTCGTGGTC-3′	
+27	rs1042714	Forward Primer	Vic- TCGTCCCTTTGCTGCGT
		5′-CCTTCTTGCTGGCACCCAAT-3′	Fam-TCGTCCCTTTCCTGCGT
		Reverse Primer	
		5′-TGCCCACCACCCACAC-3′	

### Statistical analysis

The demographics and characteristics of the 374 infants included, with either upper respiratory infection or lower respiratory tract infection, are presented as frequencies and proportions for specific categorical variables and medians and interquartile ranges were calculated for continuous variables. All analyses were stratified by race (Caucasian or African American), as a surrogate measure of European or African ancestry, to reduce the known impact of population substructure which can produce spurious associations [41]. A pairwise linkage disequilibrium (LD) was estimated for the SNPs in ADRB2 using the standardized summary statistics D’ and r, calculated by the HaploView program (Whitehead Institute for Biomedical Research, Cambridge, MA, USA). SNPs were tested for deviations from Hardy Weinberg proportions, also using Haploview [42]. The promoter, coding and combined promoter and coding SNPs were assigned to blocks using D’ confidence interval of Gabriel et al. [43]. Individual haplotypes were estimated using the program PHASE v2.1.1 [44, 45]. The haplotype frequencies for each block were estimated using the expectation maximization (EM) algorithm, again using the HaploView program. Non-parametric Kruskal-Wallis test were applied to compare the difference in BSS among infants with each SNP genotype, as well as each haplotype genotype. Haplotype genotypes with frequency less than 2 % were excluded from haplotype analysis. We used a proportional odds model for the ordinal BSS to test the additive effect of haplotypes. Covariates included in the regression model included infant age at enrollment, gender, daycare exposure, secondhand smoke exposure, any prior history of breastfeeding, any siblings at home, and enrollment season. All analyses were conducted separately according to race. All analyses were performed using R-software version 2.11.1 (www.r-project.org). We used a two-sided 5 % significance level for all statistical inferences.

## Results

There were in total 374 infants, 261 Caucasians and 113 African Americans, enrolled in the TCRI cohort with available DNA and at least one ADRB2 polymorphism tested (Table 2). The median infants’ age at enrollment was 11 weeks (interquartile range [IQR]: 6, 25). The average birth weight of these infants was 3317 g (standard deviation [SD]: 455.8). Among those infants, 207 (55 %) were male, 198 (53 %) were breastfed, 107 (29 %) attended daycare at the time of enrollment, 222 (60 %) were exposed to secondhand smoke, and 283 (76 %) had older siblings at home. Seventy six percent (*n* = 285) of infants had bronchiolitis at enrollment and 64 % (*n* = 239) were identified as RSV positive. The median bronchiolitis severity score was 5.5 (IQR: 2.5, 8.5). Compared to African American infants, Caucasian infants were more likely to have a higher birth weight, younger age at enrollment, been breast fed, have secondhand smoke exposure, have private insurance, have mothers who were older and had a higher education level, and have mothers who were less likely to have asthma. In addition, Caucasian infants were more to have lower respiratory tract infection instead of upper respiratory infection, and have more severe respiratory infections based on the BSS (*p* < 0.05).

**Table 2:** Characteristics of infants and their biological mothers (*N* = 374)

	Caucasian (*N* = 261)	African American (*N* = 113)	*P* value
Infant characteristics			
Birth weight (grams) (*N* = 372), median (IQR*)	3345 (3090, 3657)	3147 (2892, 3430)	<0.001
Gestational age (weeks) (*N* = 371), median (IQR)	39 (38,40)	39 (38,40)	0.498
Male gender, n (%)	140 (54)	67 (59)	0.313
Age at enrollment (weeks), median (IQR)	10 (6, 21)	18 (7, 35)	<0.001
Breastfeeding, n (%)	152 (58)	46 (41)	0.002
Daycare attendance, n (%)	68 (26)	39 (35)	0.096
Secondhand smoke exposure (*N* = 371), n (%)	158 (61)	64 (57)	0.486
Having siblings, n (%)	198 (76)	85 (75)	0.894
Insurance type, n (%)			
Private	111 (43)	9 (8)	<0.001
Medicare	135 (52)	101 (89)	
None	15 (6)	3 (3)	
Season of enrollment, n (%)			
2004 – 2005	42 (16)	21 (19)	0.590
2005 – 2006	76 (29)	38 (34)	
2006 – 2007	75 (29)	31 (27)	
2007 – 2008	68 (26)	23 (20)	
RSV positive	180 (69)	59 (52)	0.002
Lower respiratory tract infection vs. upper respiratory infection, n (%)	213 (82)	72 (64)	<0.001
Bronchiolitis severity score (*n* = 373), median (IQR)	6.5 (3, 9)	4 (1, 7)	<0.001
Maternal characteristics			
Maternal age at enrollment (years)	27 (22, 31)	24 (21, 27)	<0.001
Maternal education, years (*n* = 313), n (%)			
<12	42 (19)	16 (17)	0.004
12	56 (25)	41 (44)	
>12	122 (55)	36 (39)	
Maternal asthma, n (%)	50 (19)	27 (24)	0.298

*Abbreviations*: *IQR* interquartile range

Table 3 lists the allele frequencies of 5 SNPs separately for Caucasian and African Americans. In both Caucasians and African Americans, infants had slightly more T, T, and G alleles for the three promoter SNP rs1432623, rs11168068, and rs2400707, respectively. Additionally, the distributions are similar among Caucasians and African Americans. There was a significant difference between Caucasians and African Americans in the frequency distribution of the two coding SNPs. In comparison with African Americans, Caucasians were more likely to have a G allele of rs1042713 SNP, and less likely to have a C allele of rs1042714 SNP.

**Table 3:** Allele frequencies of the 5 ADRB2 SNPs (*P* value compares the frequency difference between Caucasian and African Americans)

	SNP	Caucasian			African American			*P* value
		(*N* = 261)			(*N* = 113)			
		N	Allele frequencies (%)		N	Allele frequencies (%)		
rs1432623	C/T	252	42.9	57.1	111	44.6	55.4	0.785
rs11168068	C/T	252	42.5	57.5	110	45.0	55.0	0.679
rs2400707	A/G	260	42.3	57.7	111	44.1	55.9	0.832
rs1042713	A/G	233	36.9	63.1	104	50.0	50.0	0.033
rs1042714	C/G	244	57.2	42.8	106	78.8	21.2	<0.001

**Fig. 1:**
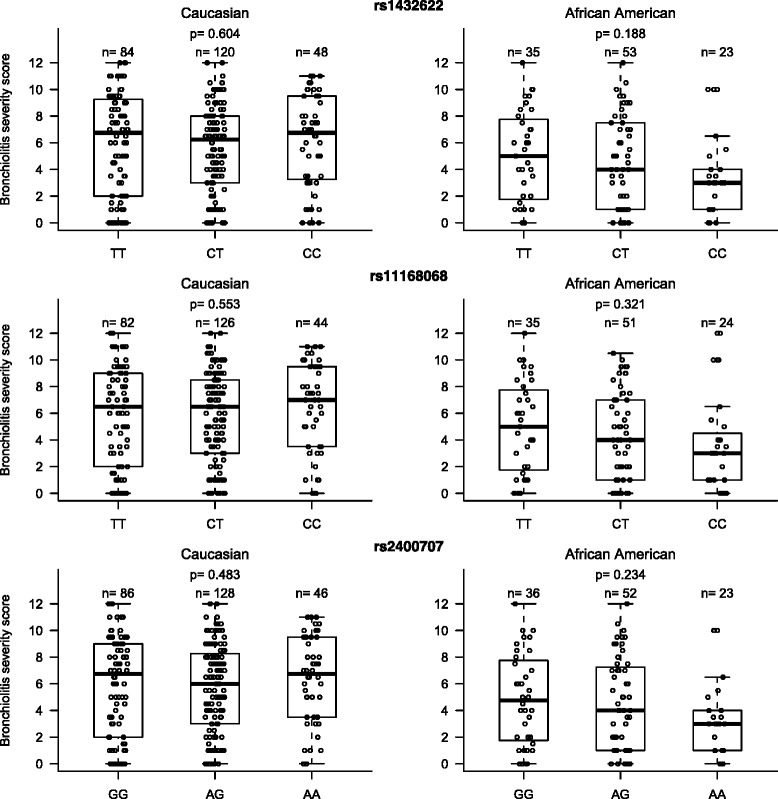
BSS distribution across each genotype of 3 promoter SNPs, stratified by race. The scatter plots of the distribution of BSS across genotypes of 3 promoter SNPs (rs1432623, rs11168068, and rs2400707) separately for Caucasian and African American infants. We further overlay box-and-whisker plot of BSS on each scatter plot. P values were obtained from non-parametric Kruskal-Wallis test

**Fig. 2:**
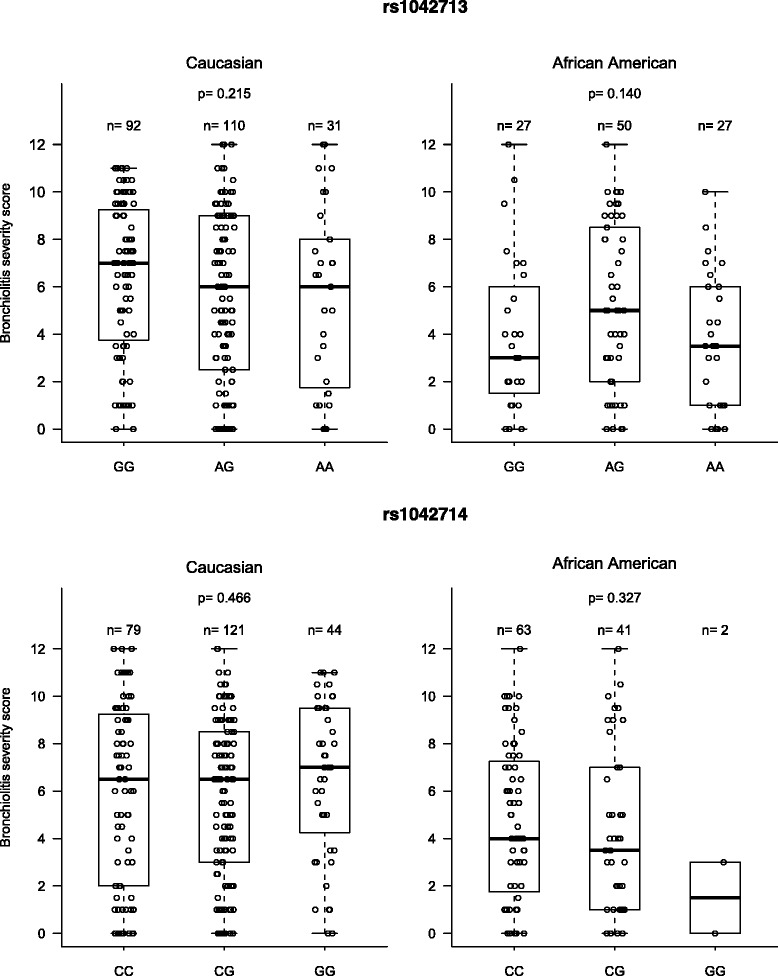
BSS distribution across each genotype of 2 coding SNPs, stratified by race. The scatter plots of the distribution of BSS across genotypes of 2 coding SNPs (rs1042713 and rs1042714) separately for Caucasian and African American infants. We further overlay box-and-whisker plot of BSS on each scatter plot. *P* values were obtained from non-parametric Kruskal-Wallis test

Within each SNP, there was no statistical difference in BSS among each genotype (Figs. 1 and 2). This non statistical difference was consistent within both Caucasians and African Americans. Among African Americans, there was a decreasing trend of the severity of acute respiratory infection with an increasing number of copies of C, C, and A allele of the three promoter SNPs rs1432623, rs11168068, and rs2400707, respectively (Fig. 1). However, analyses with additive models indicated no statistical significance (*p* > 0.05).

**Fig. 3:**
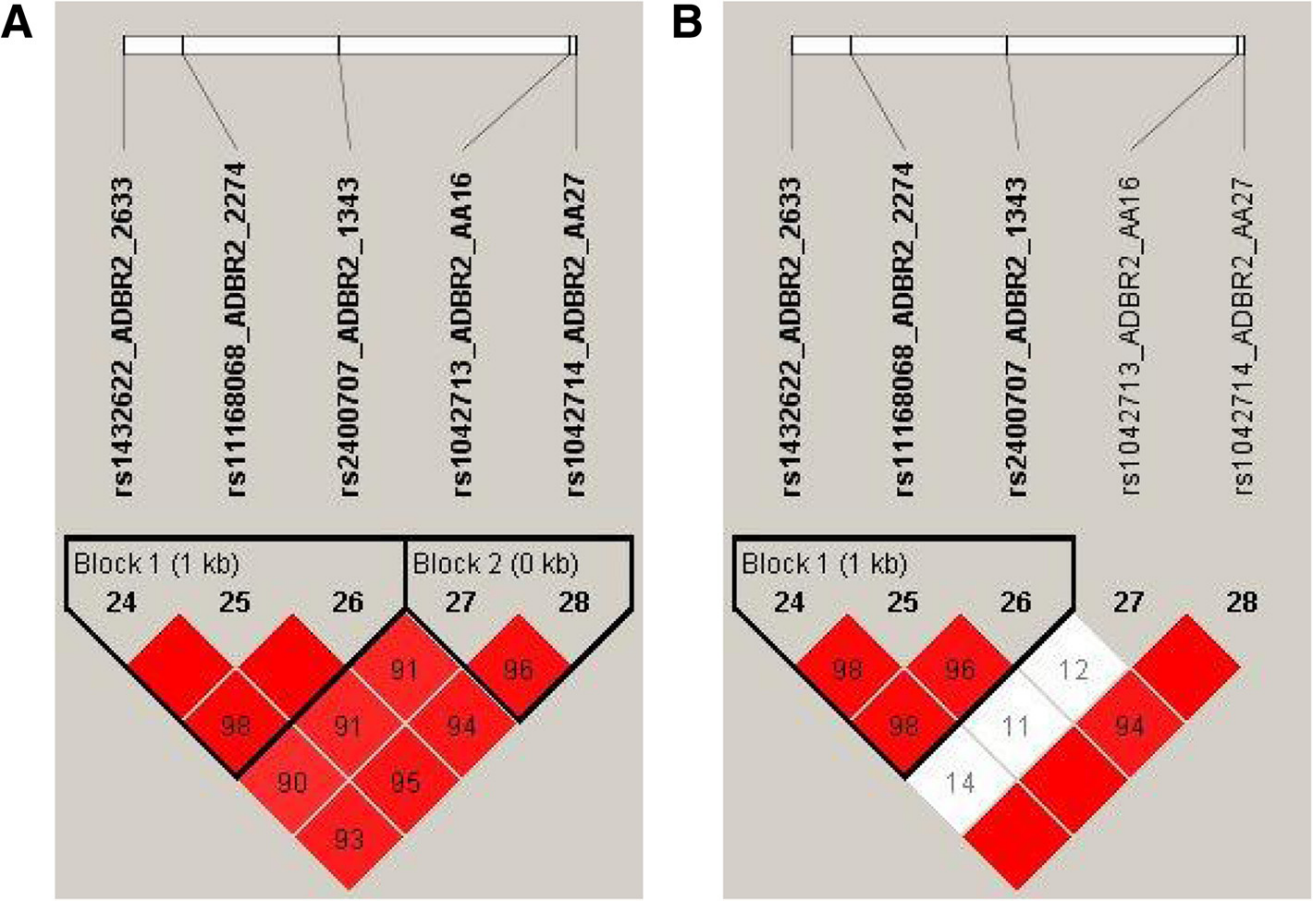
Linkage disequilibrium (LD) plot in Caucasian (**a**) and African Americans (**b**). The plot illustrates the linkage disequilibrium mapping using the HaploView program in Caucasian (**a**) and African Americans (**b**) at 5 loci of the B2-adrenergic receptor gene: -2633, -2274, -1343 (in the promoter region), and +46 and +79 (in the coding block)

Among 2^5^ (32) possible haplotype combinations, only four haplotypes with more than 2 % of frequency were identified (Table 4 and Fig. 3). There were two promoter haplotypes and three coding block haplotypes. There were no statistically significant differences in the promoter and coding block haplotype frequencies between Caucasian and African Americans. However, when all 5 SNPs were combined, the distribution of haplotype frequency was significantly different between Caucasians and African Americans, with genotype CCAAC more common in African Americans and rare in Caucasians (*p* < 0.001).

**Table 4:** Haplotype frequencies of the ADRB2 promoter and coding block SNPs (Haplotype genotypes with less than 2 % frequency were not shown in the table)

	Caucasians	African Americans	*P* value
	(*N* = 261)	(*N* = 113)	
Promoter haplotype			
CCA	42.77	44.86	0.636
TTG	56.20	53.74	
Coding block haplotype			
AC	37.40	50.47	0.622
GC	19.01	28.97	
GG	43.18	20.56	
Combined haplotype			
CCAGG	41.32	20.56	<.001
TTGAC	35.33	25.70	
TTGGC	19.63	28.04	
CCAAC	1.24	23.83

**Fig. 4:**
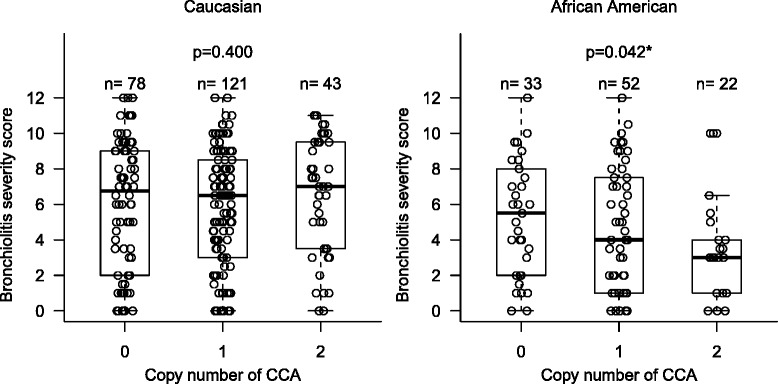
BSS distribution stratified by copy number of promoter haplotype CCA and separated by race. The scatter plots and the box-and-whisker plots of BSS across 0, 1, and 2 copies of promoter haplotype CCA for both Caucasian and African American infants. P values were obtained from multivariable regression model adjusted for infant age at enrollment, gender, daycare exposure, secondhand smoke exposure, any prior history of breastfeeding, any siblings at home, and enrollment season

Promoter haplotype CCA was associated with a decreased BSS in African Americans in a dose dependent manner (*p* = 0.037 in the univariate analysis) (Fig. 4). Infants without the CCA haplotype had the highest BSS (median 5.5, interquartile range [IQR]: 2.0, 8.0). Infants with one copy of CCA haplotype had a lower bronchiolitis severity score (median 4.0, IQR: 1.0, 7.5). Infants with 2 copies of the CCA haplotype had the comparatively lowest BSS (median 3.0, IQR: 1.0, 4.0). This dose dependent relationship persisted after adjusting for infant age, gender, daycare exposure, secondhand smoke exposure, prior history of breastfeeding, siblings at home, and enrollment season (adjusted odds ratio: 0.59, 95 % confidence interval [CI]: 0.36, 0.98, *p* = 0.042). This protective effect of CCA haplotype was consistent when we categorized the acute respiratory infection into upper respiratory infection and lower respiratory tract infection. In a similar dose dependent manner, infants with more copies of CCA haplotype were less likely to have lower respiratory tract infection compared with infants with less copies of CCA haplotype (*p* = 0.05). On the other hand, promoter haplotype TTG was associated with a higher BSS in African Americans (Additional file 1).

**Fig. 5:**
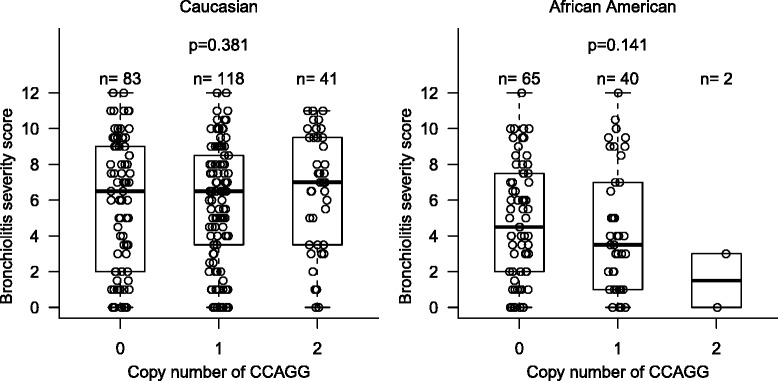
BSS distribution stratified by copy number of combined haplotype CCAGG and separated by race. The scatter plots and the box-and-whisker plots of BSS across 0, 1, and 2 copies of combined haplotype CCAGG for both Caucasian and African American infants. P values were obtained from multivariable regression model adjusted for infant age at enrollment, gender, daycare exposure, secondhand smoke exposure, any prior history of breastfeeding, any siblings at home, and enrollment season

**Fig. 6:**
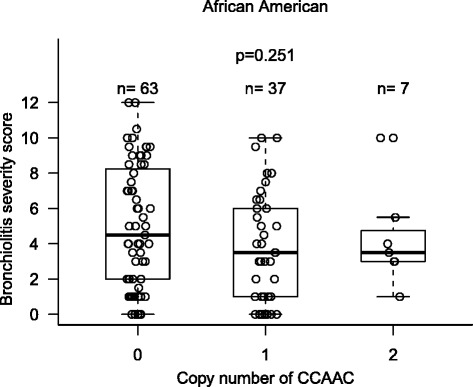
BSS distribution stratified by copy number of combined haplotype CCAAC in African Americans infants. The scatter plots and the box-and-whisker plots of BSS across 0, 1, and 2 copies of combined haplotype CCAAC for African American infants. P values were obtained from multivariable regression model adjusted for infant age at enrollment, gender, daycare exposure, secondhand smoke exposure, any prior history of breastfeeding, any siblings at home, and enrollment season

The protective effect of CCA haplotype in African Americans was identified in all combined haplotypes with CCA combination (Figs. 5 and 6). However, due to the sample size, the effect was not statistically significant. In addition, all African American infants with a coding block haplotype GG had CCA combination in their promoter region; therefore, there was a similar additive protective effect of GG in African Americans (Additional file 2). There was no relationship between BSS and the other two coding block haplotypes in African Americans (Additional files 3 and 4). This was also true for the combined haplotypes (Additional files 5 and 6).

In both Caucasian and African American infants, there were no significant relationships between BSS and coding block haplotypes except for GG haplotype among African Americans (Additional file 2). This was also true for the combined haplotypes. In general, there was no significant relationship between BSS and any haplotypes in Caucasian infants. There was no similar promoter haplotype CCA relationship with BSS in Caucasians as observed in the African Americans (Fig. 4).

## Discussion

In this study we have identified a haplotype in the promoter region of the ADRB2 gene which is associated with a decreased BSS in African American infants. This promoter haplotype of the ADRB2 gene was not associated with the severity of respiratory infections in Caucasian infants. The common coding block polymorphisms, corresponding to amino acid changes at codons 16 and 27, were not associated with a protective effect on severity of respiratory infections in Caucasian or African American infants. Our results agree with the studied conducted by Janssen and colleagues, who found no relationship between the SNP rs1042713 and susceptibility to RSV bronchiolitis in a European population [17].

This observation that the CCA promoter region haplotype is associated with a protective effect on respiratory viral infection severity is similar to our observation in a cohort of adults hospitalized for an asthma exacerbation, where this haplotype was associated with spirometric improvement in response to asthma-specific therapy that included beta-agonists and corticosteroids [34]. In that study, we noted spirometric improvement only in Caucasian patients, although the small number of African American non-responders in that cohort may have limited our ability to detect a similar effect in African Americans [34].

The mechanism by which a haplotype in the promoter region of the ADRB2 gene is associated with a decreased BSS is not known. However, we can speculate that ADRB2 promoter haplotype may alter ADRB2 expression on airway smooth muscle cells and thereby influence basal airway tone. McGraw et al. showed that SNPs in the promoter region of the ADRB2 may regulate gene expression [46]. Moore et al. demonstrated that ADRB2 genetic variants are associated with differences in ADRB2 expression in cultured human airway smooth muscle (HASM) cells, an in vitro system used as a model for changes in the human airway [47] These studies were performed prior to identification of the complex promoter haplotypes, and we are not aware that these haplotypes have been studied in HASM. Small differences in airway tone could thus be important in the setting of acute viral respiratory infections.

Although beyond the scope of the current observational study, we also speculate that ADRB2 genotype might also influence response to bronchiolitis-specific therapy. The primary goal of TCRI was to establish a longitudinal, prospective investigation of infants and their biological mothers on the acute and the long-term health consequences of varying viral respiratory tract infections on early childhood outcomes [37]. Although a BSS was assigned for each infant at acute care visit or hospital admission, we did not study or quantify the effects of different treatments, including beta-agonist therapy. A number of clinical studies have suggested that some infants with bronchiolitis appear to improve with bronchodilator therapy, while on average it is not efficacious [48–50]. As ADRB2 is the primary target of bronchodilator therapy, variation in response to beta-agonists might reflect genetic variants in ADRB2.

The diversity of SNPs of the ADRB2 gene was explored by Drysdale et al., using immortalized lymphocytes from 23 Caucasians, 19 African-Americans, 20 Asians, and 15 Hispanic-Latinos [27]. This report from 2000 included the 2 coding block SNPs (+46 and +79) and a number of promoter SNPs extending to 1023 bp upstream of the translation start site. Hawkins et al. examined a 5.3-kb region of the ADRB2 in 429 Caucasians and 240 African-Americans and identified similar minor allele frequencies in the 3 promoter SNPs (-2387, -2274, and -1343) and in the coding block SNPs discussed in this manuscript [28]. Such frequency distributions observed in our study are consistent with the allele frequencies reported in the reference population of the International HapMap Project [51] and Hawkins et al. [28]. Our stratified analyses by race therefore allow us to detect the association between severity of a respiratory illness and haplotype frequency within each racial group, and to minimize the spurious associations due to such population substructure.

We recognize that the significance of our findings is limited by our small sample size and in classifying infants in this study by a severity score rather than by measuring some direct response to bronchodilator therapy. However, BSS correlated with the severity of disease (classified as upper respiratory and lower respiratory illness) well. Our sensitivity analyses with the severity of disease (upper versus lower respiratory illness) showed consistent results indicated that BSS is a valid surrogate of disease severity. Lastly, although a p value of 0.042 is at the threshold of statistical significance, this p value reflects sample size of 113 African Americans in our study. Whether the association between BSS and CCA haplotype in African Americans is powerful or not depends upon not only p value, but also upon the effect size such as odds ratio and the clinical significance of the findings. As a difference of 0.5 in BSS is considered as clinically significant, a 65 % reduced odds of developing a more severe disease (higher BSS) comparing 2 copies of CCA with 0 copy of CCA might also be clinically significant and meaningful.

We hope that this hypothesis-generating study will encourage the incorporation of pharmacokinetic data in future studies of bronchiolitis. Although validation studies in other populations are needed, this study underscores that the development of personalized approaches to the treatment of bronchiolitis may benefit from the analysis of specific genetic variants.

## Conclusions

In summary, we have observed that promoter region haplotype of ADRB2 CCA is associated with a protective effect on respiratory illness severity in African American infants. In this era of personalized medicine, ADRB2 genotype may be predictive of the severity of respiratory viral infection and potential response to therapy for the most common acute infant illness and one for which we currently have no therapy. Next steps would be to validate these findings, as well as evaluate available clinical trial data for which DNA might be available.

## Additional files

Additional file 1:
**BSS distribution by copy number of promoter haplotype TTG and stratified by race.** The scatter plots and the box-and-whisker plots of BSS across 0, 1, and 2 copies of promoter haplotype TTG for both Caucasian and African American infants. *P* values were obtained from multivariable regression model adjusted for infant age at enrollment, gender, daycare exposure, secondhand smoke exposure, any prior history of breastfeeding, any siblings at home, and enrollment season. (PDF 17 kb) Additional file 2:
**BSS distribution stratified by copy number of coding block haplotype GG and separated by race.** The scatter plots and the box-and-whisker plots of BSS across 0, 1, and 2 copies of coding block haplotype GG for both Caucasian and African American infants. *P* values were obtained from multivariable regression model adjusted for infant age at enrollment, gender, daycare exposure, secondhand smoke exposure, any prior history of breastfeeding, any siblings at home, and enrollment season. (PDF 11 kb) Additional file 3:
**BSS distribution stratified by copy number of coding block haplotype AC and separated by race.** The scatter plots and the box-and-whisker plots of BSS across 0, 1, and 2 copies of coding block haplotype AC for both Caucasian and African American infants. *P* values were obtained from multivariable regression model adjusted for infant age at enrollment, gender, daycare exposure, secondhand smoke exposure, any prior history of breastfeeding, any siblings at home, and enrollment season. (PDF 11 kb) Additional file 4:
**BSS distribution stratified by copy number of coding block haplotype GC and separated by race.** The scatter plots and the box-and-whisker plots of BSS across 0, 1, and 2 copies of coding block haplotype GC for both Caucasian and African American infants. *P* values were obtained from multivariable regression model adjusted for infant age at enrollment, gender, daycare exposure, secondhand smoke exposure, any prior history of breastfeeding, any siblings at home, and enrollment season. (PDF 11 kb) Additional file 5:
**BSS distribution stratified by copy number of combined haplotype TTGAC and separated by race.** The scatter plots and the box-and-whisker plots of BSS across 0, 1, and 2 copies of combined haplotype TTGAC for both Caucasian and African American infants. *P* values were obtained from multivariable regression model adjusted for infant age at enrollment, gender, daycare exposure, secondhand smoke exposure, any prior history of breastfeeding, any siblings at home, and enrollment season. (PDF 11 kb) Additional file 6:
**BSS distribution stratified by copy number of combined haplotype TTGGC and separated by race.** The scatter plots and the box-and-whisker plots of BSS across 0, 1, and 2 copies of combined haplotype TTGGC for both Caucasian and African American infants. *P* values were obtained from multivariable regression model adjusted for infant age at enrollment, gender, daycare exposure, secondhand smoke exposure, any prior history of breastfeeding, any siblings at home, and enrollment season. (PDF 11 kb)
