# Supplementary figures and images for: β2-Adrenergic receptor promoter haplotype influences the severity of acute viral respiratory tract infection during infancy: a prospective cohort study
Source: BMC Med Genet. 2015 Sep 14;16:82. doi: 10.1186/s12881-015-0229-3 (PMC4570703; doi:10.1186/s12881-015-0229-3)

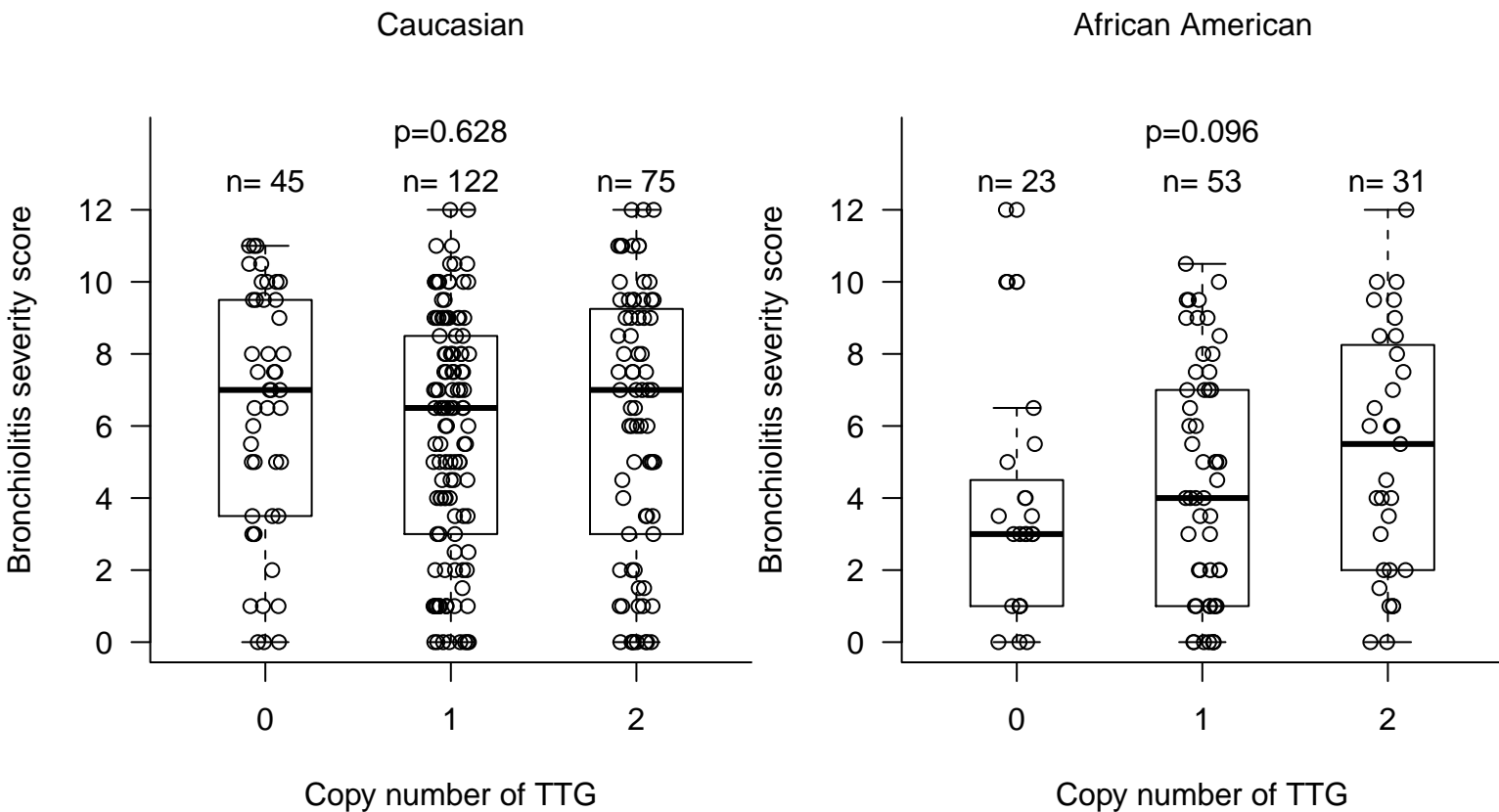

Supplement: Additional file 1: — BSS distribution by copy number of promoter haplotype TTG and stratified by race. The scatter plots and the box-and-whisker plots of BSS across 0, 1, and 2 copies of promoter haplotype TTG for both Caucasian and African American infants. P values were obtained from multivariable regression model adjusted for infant age at enrollment, gender, daycare exposure, secondhand smoke exposure, any prior history of breastfeeding, any siblings at home, and enrollment season. (PDF 17 kb) [file 12881_2015_229_MOESM1_ESM.pdf]

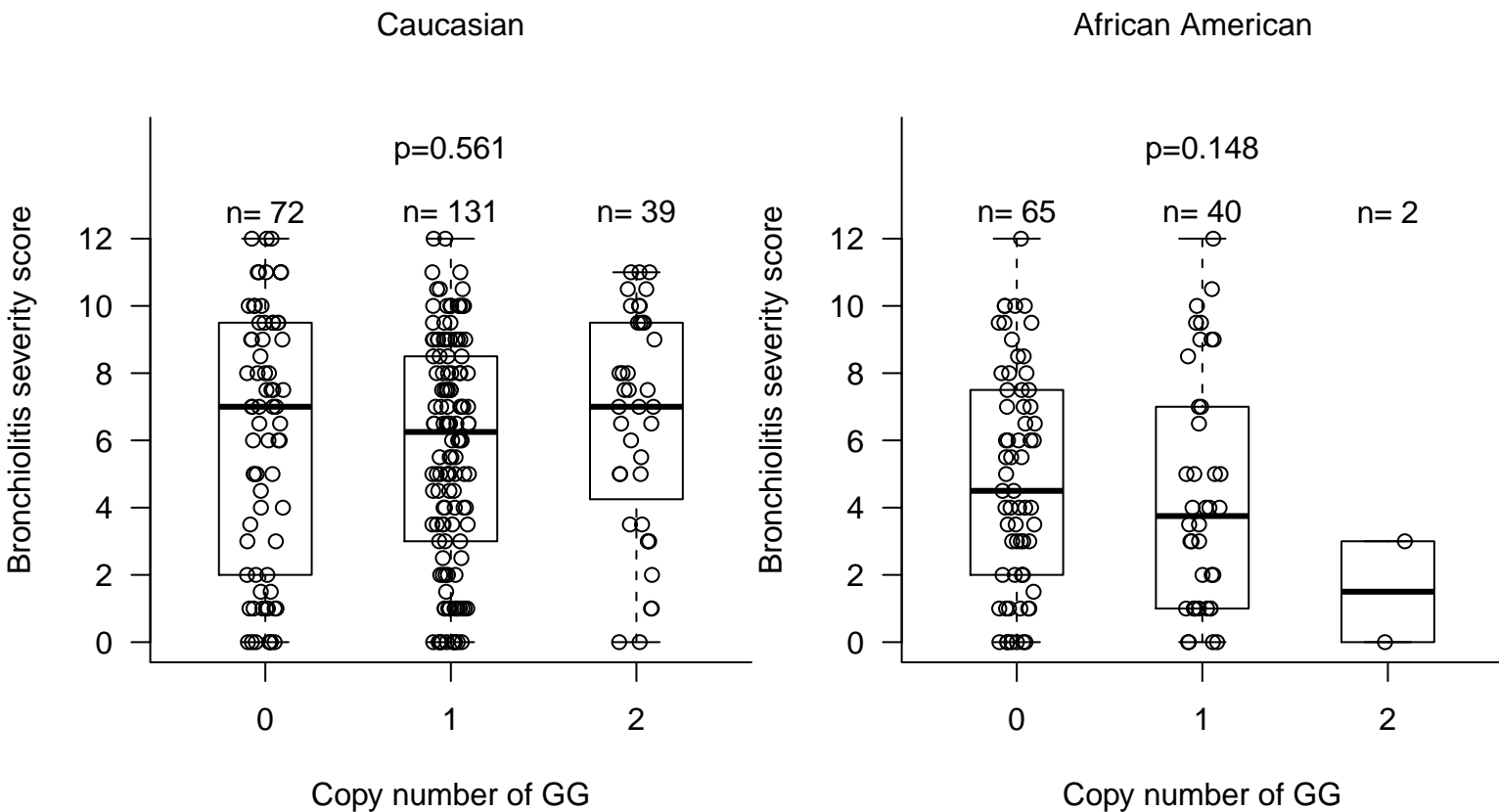

Supplement: Additional file 2: — BSS distribution stratified by copy number of coding block haplotype GG and separated by race. The scatter plots and the box-and-whisker plots of BSS across 0, 1, and 2 copies of coding block haplotype GG for both Caucasian and African American infants. P values were obtained from multivariable regression model adjusted for infant age at enrollment, gender, daycare exposure, secondhand smoke exposure, any prior history of breastfeeding, any siblings at home, and enrollment season. (PDF 11 kb) [file 12881_2015_229_MOESM2_ESM.pdf]

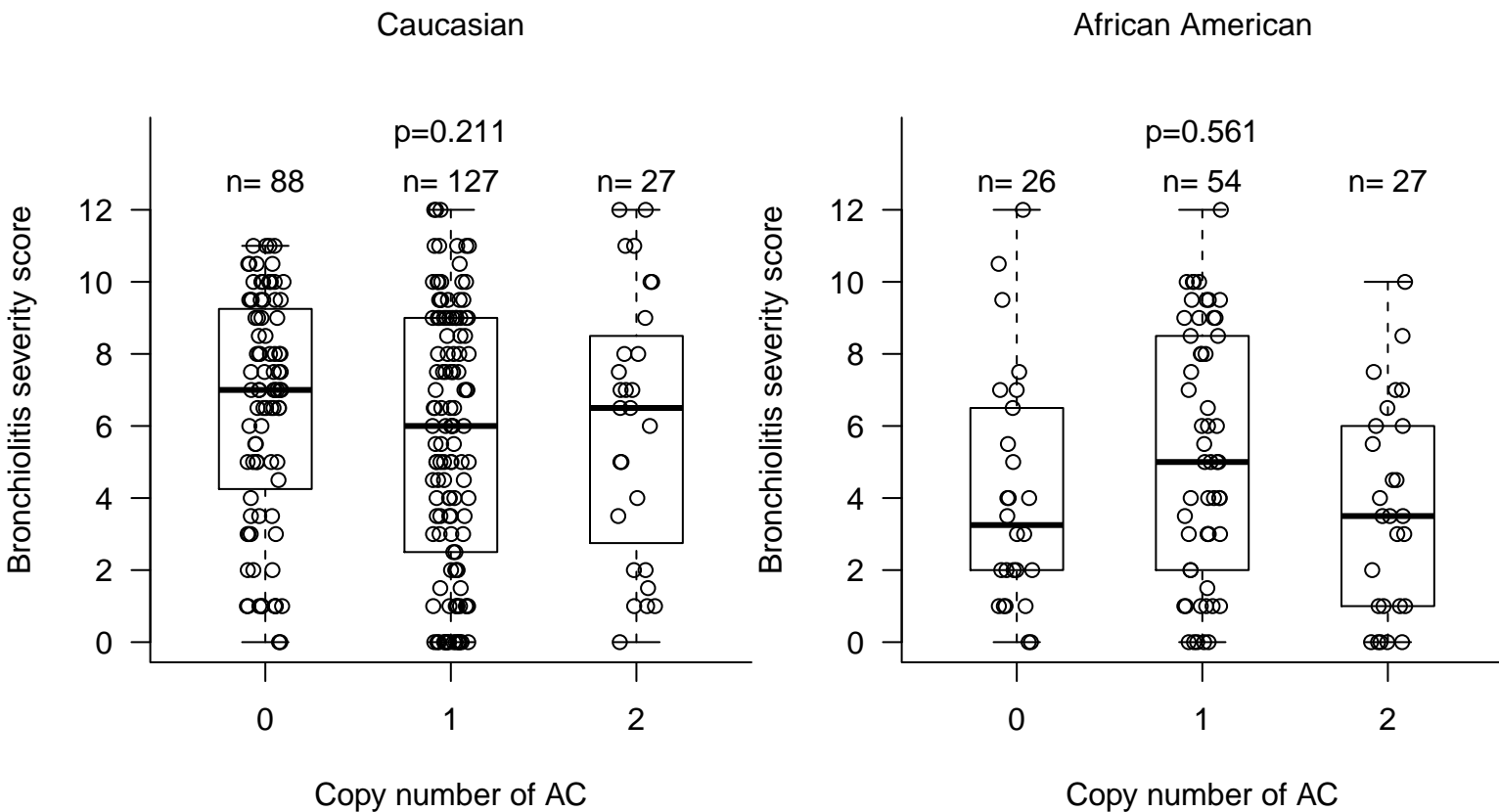

Supplement: Additional file 3: — BSS distribution stratified by copy number of coding block haplotype AC and separated by race. The scatter plots and the box-and-whisker plots of BSS across 0, 1, and 2 copies of coding block haplotype AC for both Caucasian and African American infants. P values were obtained from multivariable regression model adjusted for infant age at enrollment, gender, daycare exposure, secondhand smoke exposure, any prior history of breastfeeding, any siblings at home, and enrollment season. (PDF 11 kb) [file 12881_2015_229_MOESM3_ESM.pdf]

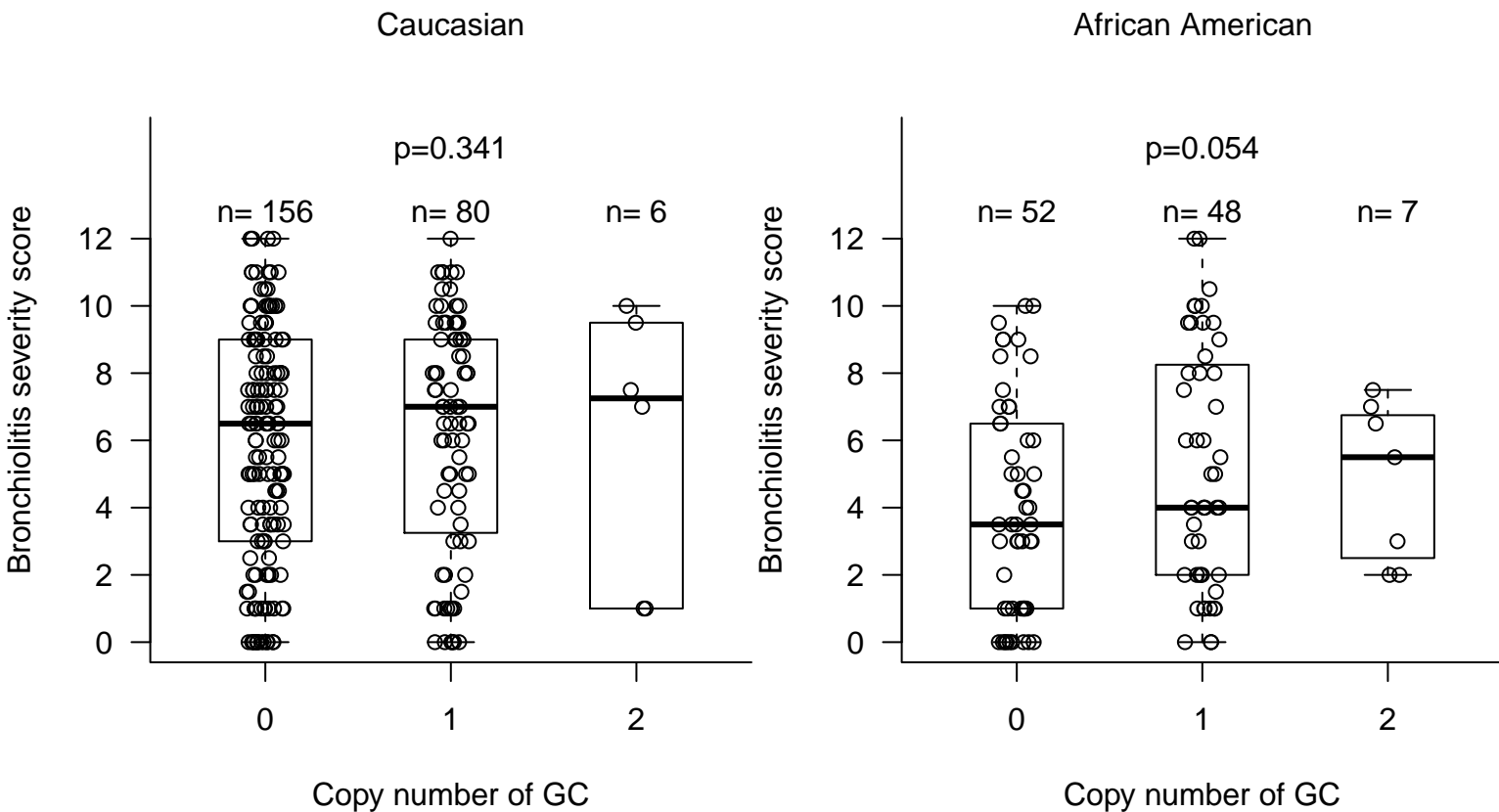

Supplement: Additional file 4: — BSS distribution stratified by copy number of coding block haplotype GC and separated by race. The scatter plots and the box-and-whisker plots of BSS across 0, 1, and 2 copies of coding block haplotype GC for both Caucasian and African American infants. P values were obtained from multivariable regression model adjusted for infant age at enrollment, gender, daycare exposure, secondhand smoke exposure, any prior history of breastfeeding, any siblings at home, and enrollment season. (PDF 11 kb) [file 12881_2015_229_MOESM4_ESM.pdf]

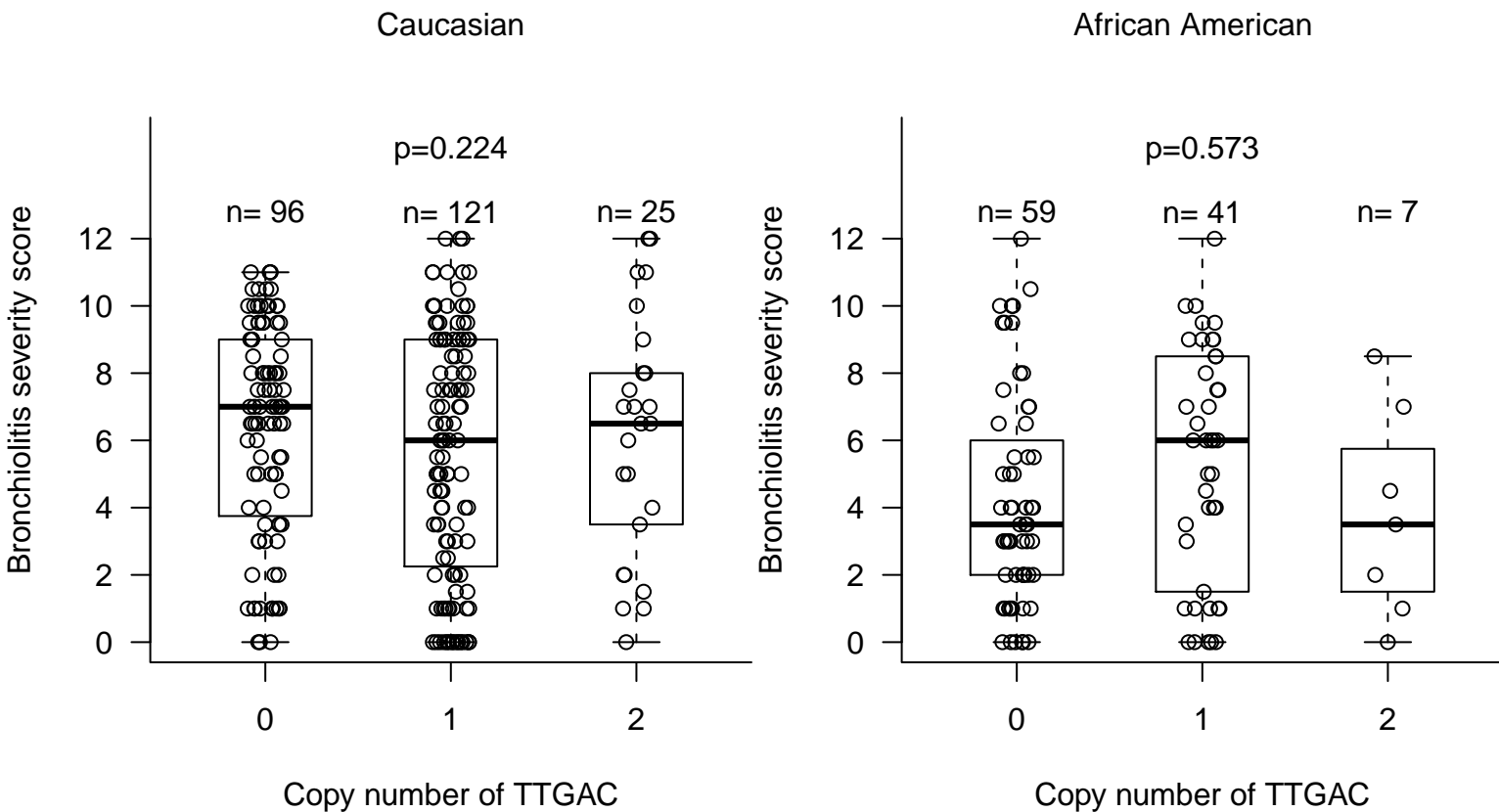

Supplement: Additional file 5: — BSS distribution stratified by copy number of combined haplotype TTGAC and separated by race. The scatter plots and the box-and-whisker plots of BSS across 0, 1, and 2 copies of combined haplotype TTGAC for both Caucasian and African American infants. P values were obtained from multivariable regression model adjusted for infant age at enrollment, gender, daycare exposure, secondhand smoke exposure, any prior history of breastfeeding, any siblings at home, and enrollment season. (PDF 11 kb) [file 12881_2015_229_MOESM5_ESM.pdf]

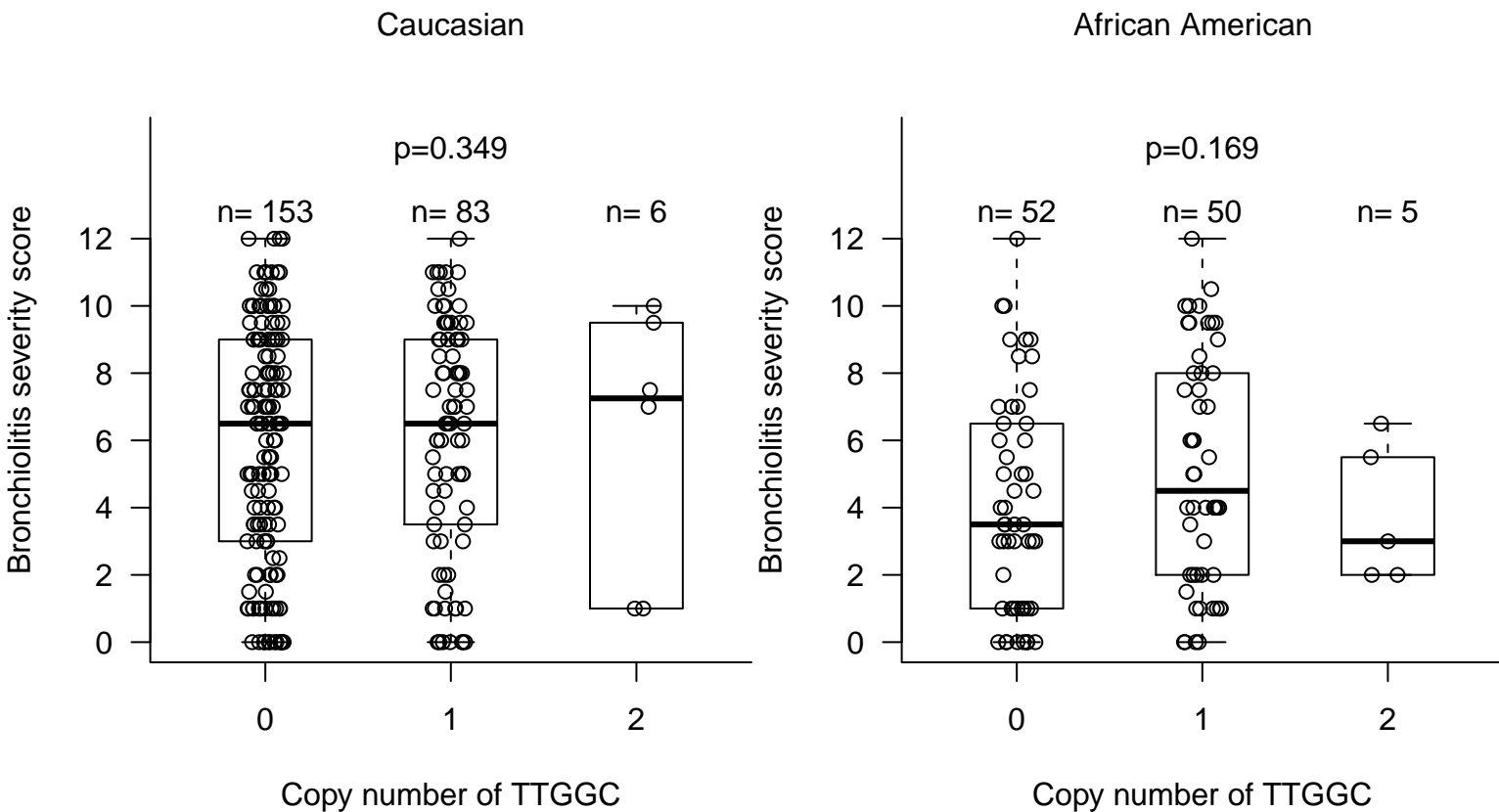

Supplement: Additional file 6: — BSS distribution stratified by copy number of combined haplotype TTGGC and separated by race. The scatter plots and the box-and-whisker plots of BSS across 0, 1, and 2 copies of combined haplotype TTGGC for both Caucasian and African American infants. P values were obtained from multivariable regression model adjusted for infant age at enrollment, gender, daycare exposure, secondhand smoke exposure, any prior history of breastfeeding, any siblings at home, and enrollment season. (PDF 11 kb) [file 12881_2015_229_MOESM6_ESM.pdf]
